# Transcriptional Changes Involved in Atrophying Muscles during Prolonged Fasting in Rats

**DOI:** 10.3390/ijms21175984

**Published:** 2020-08-20

**Authors:** Marianne Ibrahim, Thierry Wasselin, Etienne Challet, Alain Van Dorsselaer, Yvon Le Maho, Thierry Raclot, Fabrice Bertile

**Affiliations:** 1 Institut Pluridisciplinaire Hubert Curien (IPHC), CNRS, Université de Strasbourg, 67000 Strasbourg, France; marianneibrahim@hotmail.com (M.I.); thierry.wasselin@med.uni-goettingen.de (T.W.); vandors@unistra.fr (A.V.D.); yvon.lemaho@iphc.cnrs.fr (Y.L.M.); thierry.raclot@iphc.cnrs.fr (T.R.); 2 Laboratoire de Spectrométrie de Masse Bio-Organique, 25 rue Becquerel, F-67087 Strasbourg, France; 3 Institute of Cellular and Integrative Neurosciences, CNRS, Université de Strasbourg, F-67000 Strasbourg, France; challet@inci-cnrs.unistra.fr; 4 Département Ecologie, Physiologie, Ethologie, 23 rue Becquerel, F-67087 Strasbourg, France; 5 Centre Scientifique de Monaco, 8 quai Antoine 1er, 98000 Monaco, Monaco

**Keywords:** fasting, transcriptomics, rat, muscle, protein breakdown, protein synthesis, oxidative stress, fuel metabolism

## Abstract

Food deprivation resulting in muscle atrophy may be detrimental to health. To better understand how muscle mass is regulated during such a nutritional challenge, the current study deciphered muscle responses during phase 2 (P2, protein sparing) and phase 3 (P3, protein mobilization) of prolonged fasting in rats. This was done using transcriptomics analysis and a series of biochemistry measurements. The main findings highlight changes for plasma catabolic and anabolic stimuli, as well as for muscle transcriptome, energy metabolism, and oxidative stress. Changes were generally consistent with the intense use of lipids as fuels during P2. They also reflected increased muscle protein degradation and repressed synthesis, in a more marked manner during P3 than P2 compared to the fed state. Nevertheless, several unexpected changes appeared to be in favor of muscle protein synthesis during fasting, notably at the level of the phosphatidylinositol-3-kinase (PI3K)/protein kinase B (AKT)/mammalian target of rapamycin (mTOR) signaling pathway, transcription and translation processes, and the response to oxidative stress. Such mechanisms might promote protein sparing during P2 and prepare the restoration of the protein compartment during P3 in anticipation of food intake for optimizing the effects of an upcoming refeeding, thereby promoting body maintenance and survival. Future studies should examine relevance of such targets for improving nitrogen balance during catabolic diseases.

## 1. Introduction

There is obvious evidence that cardiac and skeletal muscles are of primary importance for well-being, metabolic health maintenance, and healthy ageing [1,2,3,4]. In one hand, the innate ability of the heart to contract ensures continuous supply of oxygen and nutrients to the organs. On the other hand, skeletal muscle is the largest organ in the body, comprising 40% of total body weight in humans [5], and contractile properties of its fibers are responsible for an individual’s ability to breathe, maintain posture, or move and accomplish movements. To produce energy and contraction, skeletal muscle uses either lipid fuels or carbohydrates, muscle glycogen stores being mobilized when the availability of dietary glucose decreases [6]. Skeletal muscles can also be seen as a reservoir of amino acids, whose mobilization can serve for protein synthesis in various organs [1], and for liver glucose neosynthesis to ensure maintenance of glycemia during food deprivation [7]. In addition, a significant thermogenic role for skeletal muscle has been strongly suggested [8]. Finally, skeletal muscle exerts a number of endocrine or paracrine effects on various physiological processes through the production of the so-called myokines [9]. Therefore, bodily functions of muscle make its maintenance crucial for the organism’s homeostasis. However, muscle integrity is inevitably challenged throughout life. Muscle atrophy occurs during ageing [10], within certain diseases [11], and in situations of disuse and/or microgravity [12] or fasting [13,14]. In addition, muscle atrophy has already been associated with the development of metabolic diseases [15]. However, there is virtually no fully effective countermeasure today to fight the loss of muscle mass.

To allow an efficient treatment against muscle atrophy to be developed, we need to understand in depth how muscle mass is regulated in wasting conditions. Prolonged fasting offers a unique model where total food deprivation triggers profound changes in the metabolic status of individuals [16,17,18]. After a short period of adaptation (phase 1) characterized by exhaustion of glycogen stores, energy needs are essentially met during phase 2 (P2) by the use of lipid fuels and the increased production of ketone bodies, while the breakdown of body proteins is reduced—the so-called protein sparing. In late fasting, before fat stores are exhausted, body proteins are increasingly mobilized for energy purposes during phase 3 (P3). Both cardiac and skeletal muscle mass and protein content are thus gradually decreased during P2 and then P3 of fasting compared to the fed state [13,19,20]. Compared to values recorded in fed animals, it has been shown that absolute and fractional synthesis rates are decreased in the heart and skeletal muscle of rats during P2 and even more during P3 of fasting, whereas at the same time whole-body protein degradation is lowered [13]. Measurements in perfused skeletal muscle from fed and prolonged fasted rats have revealed an elevation of muscle degradation that occurs essentially during P3 [21,22]. However, the molecular regulations that control the balance between protein synthesis and degradation during P2 and P3 of fasting versus fed state have only been partly investigated using Northern blot analysis. Analysis of various mRNA levels in rat gastrocnemius muscle has revealed a selective induction in P2 and coordinated upregulation in P3 for factors of the main proteolytic systems [19]. It was later shown that upregulation of gene expression for few components of the proteolytic systems during P2 and P3 of fasting affects in particular the gastrocnemius, tibialis anterior (TIB), and diaphragm muscles, whereas soleus (SOL) and heart muscles appear less affected [20]. Finally, cathepsin B, H, and L have been hypothesized to play a role in the curtailment of muscle proteolysis during P2 of fasting, as their activity levels are decreased in hindlimb muscles during prolonged fasting in both P2 and P3 rats compared to fed animals [23]. Overall, these data provide a partial view of how muscle protein balance is regulated during prolonged fasting. Deciphering of the complex regulatory networks regulating muscle protein synthesis and degradation during prolonged fasting therefore requires a more detailed analysis.

Muscle cell metabolism is intricately linked to the regulation of muscle mass, and multiple pathways and processes modulate muscle protein synthesis and breakdown [24,25,26,27,28,29,30]. It is widely recognized that signaling pathways and metabolic adaptations are orchestrated by posttranslational modifications. However, changes at the transcriptional level could help fine-tune the response an organism may exhibit when facing challenging conditions. In the context of the metabolic phases of prolonged fasting, we questioned whether transcriptional regulation could reflect changes in fuel metabolism and muscle protein/mass loss. Therefore, the major aim of this study was to analyze in-depth muscle transcriptome of fed and prolonged fasted rats. These data, together with the measurement of other metabolic parameters, enabled us to draw a detailed scheme of the molecular mechanisms modified in atrophying muscles during prolonged fasting.

## 2. Results

### 2.1. Animal Metabolic Status

Animals in the fed, P2, and P3 states (*N* = 5/group) exhibited very different metabolic profiles (Table 1). Total consumption of O_2_ was significantly decreased during fasting in both P2 and P3 rats (−23%). From similar initial values, a significant decrease was recorded in the body mass of P2 versus fed rats (−23%), and P3 versus P2 rats (−16%). The rate of body mass loss (dm/m.dt) was significantly lower (twofold) for P2 than P3 rats. The utilization of lipids as the main energy substrates during P2 was corroborated by significantly higher levels of plasma non-esterified fatty acids (NEFA) compared to values in the fed state (+37%) and a respiratory quotient (RQ) value decreased to 0.69. On the reverse, the reduced mobilization of lipid fuels during P3 resulted in a threefold reduction of plasma NEFA levels relative to the fed state, while RQ reached a value of 0.75, i.e., higher compared to that during P2. It has already been shown that body proteins are spared during P2, whereas increased loss of body proteins occurs during P3 [13,19,20,31]. Plasma levels of urea, a main product of protein degradation, nicely reflected body protein metabolism, as they tended to decrease in P2 versus fed rats and significantly reached twofold higher values during P3. Finally, plasma glucose levels were significantly lower in both fasted groups than in the fed state (−22 to 31%).

Muscles are a major reservoir for body proteins. Because of the late increase in body protein breakdown during P3 of fasting, the mass of TIB and heart muscles were significantly decreased in P3 rats versus fed or P2 animals (up to −30%). Although significance was not reached, a 15% decrease in the mass of SOL was also observed in both P2 and P3 of fasting compared to the fed state.

### 2.2. Changes in Plasma Levels of Hormones and Growth Factors in Response to Prolonged Fasting

**Figure 1:**
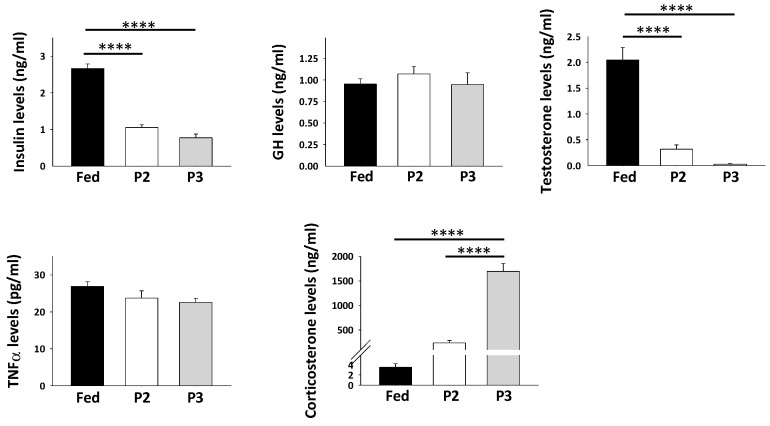
Plasma levels of hormones and growth factors (means ± SEM, *N* = 5/group). **** *p*-value < 0.0001 (one-way ANOVA and Tukey tests).

Circulating concentrations of hormones and growth factors are shown in Figure 1. Concentrations of plasma insulin and testosterone were significantly decreased during P2 (2.5- and 6.4-fold, respectively) and P3 (3.4- and 72-fold, respectively) of fasting compared to the fed state. Relative to values in fed animals, higher levels were clearly observed for corticosterone in the plasma of rats during both P2 (67-fold) and P3 (485-fold) of prolonged fasting, although significance was reached only in P3. The levels of plasma growth hormone (GH) and tumor necrosis factor alpha (TNFα) remained unchanged in fasted rats versus fed animals.

### 2.3. Overall Changes in TIB Muscle Transcriptome in Response to Prolonged Fasting

After elimination of reference genes, signal intensities from 26,535 probes were considered for microarray analysis based on three biological replicates per group (Table S1). Statistical analysis highlighted significantly changed levels for 2886 probes (ANOVA *p*-value < 0.001), which corresponded to a very low false discovery rate of 0.044 (Benjamini–Hochberg). Post hoc pairwise comparisons (Tukey adjusted *p*-value < 0.001) revealed that P3 of fasting induced changes for 2689 of them (i.e., 93%) and P2 of fasting for only 664 of them (i.e., 26%). This reveals a sharp difference in the transcriptomic response of rat tissues to prolonged fasting. Indeed, we have previously shown using similar microarray platforms that P3 elicited changes for approximately 80% but P2 for approximately 50% of the significantly changed probe signals in the liver and white adipose tissue of comparable rats [32,33]. Differences between rat groups are nicely visualized using hierarchical clustering analysis of genes and arrays that were carried out for the set of differential probe signals (Figure S1). Hence, clustering data not only showed the low variability between biological replicates, but also clearly highlighted that mRNA expression profiles in fed animals are well distinguished from those of both the P2 and P3 fasting stages in the liver and white adipose tissue [32,33], whereas P2 appeared closer to the fed state than to P3 in skeletal muscle (Figure S1).

From Table S1, it can be seen that 362 probes exhibited a significantly upregulated intensity in the TIB from P2 compared to fed animals. Intensity levels for 162 of these 362 probes were then further increased during P3. On the reverse, we observed that signal intensities were significantly reduced for 262 probes in the TIB from P2 compared to fed animals, and intensity was further reduced during P3 versus P2 for 137 of them. In addition, signal intensities from 263 probes were significantly and similarly altered in both P2 and P3 versus the fed state, with 108 of them being downregulated by fasting and 155 being upregulated. Finally, signal intensities for 2098 probes were significantly changed in P3 (not in P2) versus the fed state, with 928 of them being downregulated and 1170 being upregulated. Significant changes in the levels of four transcripts was further validated using Northern blots. The same changes were found when comparing microarrays and Northern blots for heme oxygenase 1 (*Hmox1*) and selenium-dependent glutathione peroxidase (*Gpx1*) mRNA levels in rat TIB, whereas significant upregulation of TIB superoxide dismutase 1 (*Sod1*) and catalase (*Cat*) mRNA levels observed from Northern blots was paralleled by non-significant trends toward upregulation for mRNA levels (especially *Cat*) measured from microarray analysis (see below).

Functional annotation analysis of TIB transcriptomic data revealed that differences between fed, P2, and P3 rats mainly involved transcripts known to play a role in muscle intermediary metabolism and the control of the protein balance (Figure S1). The higher fold enrichment value was notably calculated for the broad function “protein metabolism”, and the higher number of enriched Gene Ontology (GO) terms was obtained for the “mitochondrion function”.

### 2.4. Changes in the TIB Transcriptome Related to Regulation of Muscle Protein Balance

A large number of transcripts coding proteins involved in the control of muscle protein balance were found differentially regulated in TIB from fasted versus fed rats (Table S1). To give a detailed view of such changes, we distributed transcripts among different “functional groups”.

**Figure 2:**
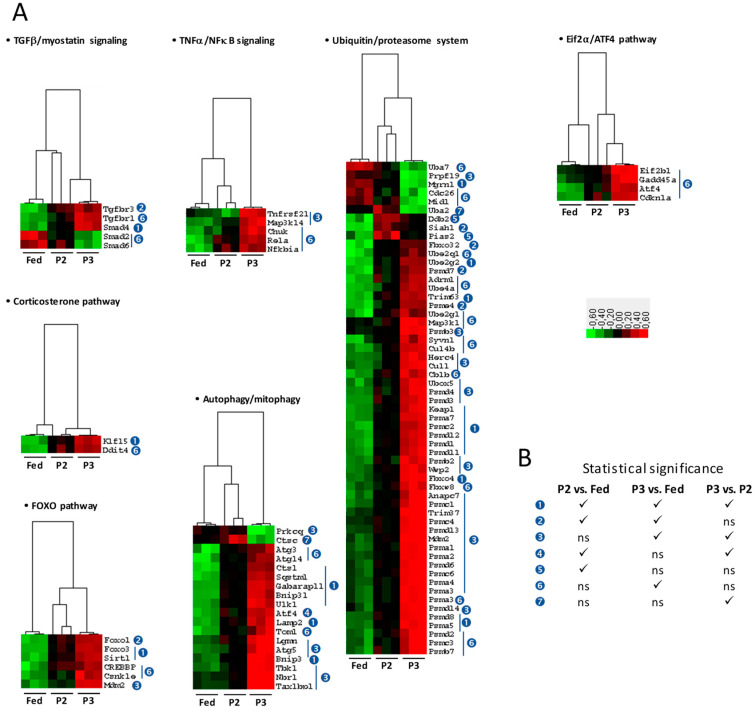
Changes related to protein degradation signaling in tibialis anterior muscle and plasma of rats during prolonged fasting. (**A**) Heatmaps showing differentially abundant transcripts between fed, phase 2 (P2), and phase 3 (P3) animals (*N* = 3/group). Red, black, and green boxes represent downregulated, intermediate, and upregulated genes, respectively. (**B**) Legend for numbers in blue circles, which indicate when mRNA levels were significantly changed (ANOVA and Tukey *p*-values < 0.001).

For muscle protein degradation-related changes (Figure 2A), functional groups included the signaling pathways for transforming growth factor beta (TGFβ) and myostatin, tumor necrosis factor alpha (TNFα), and corticosterone. Different pathways and processes were also considered, including the nuclear factor-kappa B (NFκB) pathway, the forkhead box protein O (FOXO) pathway, and the autophagy and ubiquitin–proteasome systems. The number of significant changes was low during P2 of fasting versus the fed state. They essentially included upregulations for transforming growth factor beta receptor type 3 (*Tgfbr3*), mothers against decapentaplegic homolog 4 (*Smad4*), Kruppel-like transcription factor (*Klf15*), forkhead box protein O1 and O3 (*Foxo1* and *Foxo3*), NAD-dependent protein deacetylase sirtuin-1 (*Sirt1*), AN1-type zinc finger protein 5 (*Zfand5*), and several factors that play a role in the autophagy process (*Bnip3*, *Bnip3l*, *Ctsl*, *Gabarapl1*, *Lamp2*, *Sqstm1*, *Ulk1*) or the ubiquitin–proteasome system (e.g., the E3 ubiquitin ligases *Fbxo32*, *Trim63*, *Fbxo4*, *Mgrn1*, *Pias2*, and *Siah1*, together with several proteasome subunits). Interestingly, gene expression of mitogen-activated protein kinase kinase 6 (*Map2k6*) was downregulated during prolonged fasting. These changes were generally maintained but most often exacerbated during P3 of fasting, which was also characterized by a high number of additional changes. For example, transforming growth factor beta receptor type 1 (*Tgfbr1*), dual specificity mitogen-activated protein kinase kinase 3 (*Map2k3*), cyclic AMP-dependent transcription factor ATF4 (*Atf4*), growth arrest and DNA damage-inducible protein GADD45 alpha (*Gadd45a*), and various factors of TNFα/NFκB signaling (*Map3k14*, *Tnfrsf21*, *Nfkbia*, *Rela*, *Chuk*, *Cnsk2a2*), corticosterone pathway (*Ddit4*), and autophagy (e.g., *Atg3*, *Atg5*, *Atg14*, *Tom1*, *Tax1bp1*) and ubiquitin–proteasome (e.g., *Ube2ql*, *Ube4a*, *Ube2gl*, *Map3kl*, *Psmb3*, *Psmd4*, *Psma3*) systems were found expressed at higher levels in P3 rats versus fed animals. The reverse was observed for mothers against decapentaplegic homologs 2 and 6 (*Smad2* and *Smad6*). Concerning the FOXO pathway, the levels for transcripts coding protein Mdm2 (*Mdm2*), casein kinase 1 epsilon (*Csnk1e*), and CREB-binding protein (*Crebbp*) were higher in P3 relative to the fed state.

**Figure 3:**
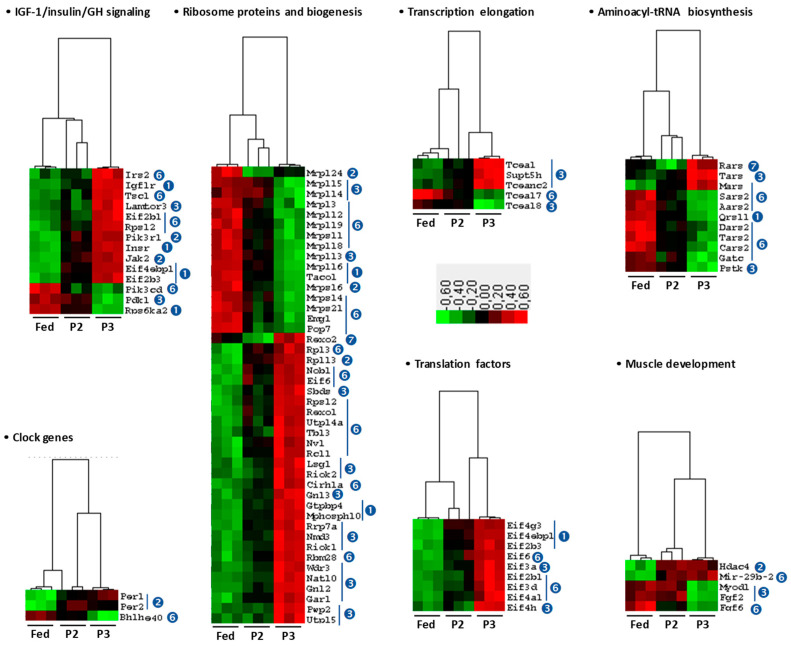
Changes related to protein synthesis signaling in tibialis anterior muscle and plasma of rats during prolonged fasting. Heatmaps showing differentially abundant transcripts between fed, phase 2 (P2), and phase 3 (P3) animals (*N* = 3/group). Red, black, and green boxes represent downregulated, intermediate, and upregulated genes, respectively. Legend for numbers in blue circles, which indicate when mRNA levels were significantly changed (ANOVA and Tukey *p*-values < 0.001), is given in Figure 2B.

For muscle protein synthesis-related changes (Figure 3), functional groups included the signaling pathways for insulin-like growth factor-1 (IGF-1) and insulin and growth hormone (GH), as well as ribosome proteins and biogenesis, transcription elongation factors, translation factors, the factors involved in aminoacyl-tRNA biosynthesis and muscle development, and finally clock genes. During P2 compared to the fed state, the vast majority of transcripts remained unchanged, except for several factors of the IGF-1/Insulin/GH signaling, with the abundance of insulin-like growth factor 1 receptor (*Igf1r*), insulin receptor (*Insr*), tyrosine-protein kinase JAK2 (*Jak2*), phosphatidylinositol 3-kinase regulatory subunit alpha (*Pik3r1*), and two clock genes (*Per1* and *Per2*) being increased. The levels of transcripts coding ribosomal proteins (*Mrps16*, *Mrpl16*, *Mrpl24*, *Rps6ka2*), translational activator of cytochrome c oxidase 1 (*Taco1*), and glutamyl-tRNA(Gln) amidotransferase subunit A (*Qrsl1*) were found to be significantly decreased during P2, and the reverse was observed for two factors involved in the processing of pre-mRNAs (*Gtpbp4*, *Mphosph10*) and histone deacetylase 4 (*Hdac4*). Increased levels were recorded during P2 of fasting for 3 translation factors (*Eif2b3*, *Eif4ebp1*, and *Eif4g3*), as well as significantly decreased levels for *Rps6ka2*. Contrary to P2, many changes occurred during P3 of fasting. First, those significant changes observed during P2 versus the fed state were either maintained or exacerbated during P3. In addition, lower levels were observed during P3 of fasting for pyruvate dehydrogenase kinase, isozyme 1 (*Pdk1*) phosphatidylinositol 3-kinase catalytic delta polypeptide (*Pik3cd*), and one clock-related mRNA (*Bhlhe40* or *Dec1*), whereas insulin receptor substrate 2 (*Irs2*), ragulator complex protein LAMTOR3 (*Lamtor3*), and harmatin (*Tsc1*) were significantly increased. Concerning factors involved in aminoacyl-tRNA biosynthesis, the levels were essentially lower during P3 (*Aars2*, *Cars2*, *Dars2*, *Gatc*, *Pstk*, *Sars2*, *Tars2*), except for methionine-tRNA ligase (*Mars*) and threonine-tRNA ligase 1 (*Tars*), which were increased. Two transcription elongation factors (*Tceal7*, *Tceal8*) were downregulated during P3 of fasting, and three others (*Tcea1*, *Supt5h*, *Tceanc2*) were upregulated. Transcripts for most of mitochondrial ribosomal proteins were expressed at significantly lower levels during P3 versus the fed state, and the reverse was true for transcripts coding cytosolic ribosomal proteins. Translation factors (*Eif2b1*, *Eif3a*, *Eif3d*, *Eif4a1*, *Eif4h*, *Eif6*) were expressed at higher levels during P3, as well as the miRNA miR-29b-2. The levels of fibroblast growth factor 2 (*Fgf2*) and 6 (*Fgf6*), as well as of myoblast determination protein 1 (*Myod1*) were significantly reduced in P3 versus fed animals.

Finally, it can be noticed that significance was not reached for changes in the levels of mRNAs that are key to the control of muscle protein balance. This was the case for proteolysis-related mRNAs such as growth/differentiation factor 8, also named myostatin (*Mstn*), and its receptors (*Acvr1b* and *Acvr2b*); TGFβ receptor (*Tgfbr2*); activin receptors (*Acvr1*, *Acvr1b*, *Acvr2a*, *Acvr2b*); androgen receptor (*Ar*); glucocorticoid receptor (*Nr3c1*); serine/threonine-protein kinase (*Sgk1*); TNF receptor-associated factor 6 (*Traf6*); and several factors of the mitogen-activated protein (MAP) kinase pathway (*Map3k7* or *Tak1*, *Mapk11*, *Mapk12*, *Mapk13*, *Mapk14*); nuclear factor NF-kappa-B p105 subunit (*Nfkb1*); and nuclear factor of kappa light polypeptide gene enhancer in B-cells 2, p49/p100 (*Nfkb2*). The same observation applies to a number of mRNAs involved in protein synthesis such as serine/threonine-protein kinase mTOR; regulatory-associated protein of mTOR complex 1 (*Rptor*); protein kinase B alpha (*Akt1*), beta (*Akt2*), and gamma (*Akt3*); glycogen synthase kinase 3 beta (*Gsk3*β); ribosomal protein S6 kinase beta-1 (*Rps6kb1*); ribosomal protein L31 (*Rpl31*); eukaryotic translation initiation factor 2 subunit 1 (*Eif2s1*); translation initiation factor eIF-2B subunit epsilon (*Eif2b5*); eukaryotic translation initiation factor 4E (*Eif4e*); growth hormone receptor (*Ghr*); myogenin (*Myog*); and fibroblast growth factor 4 (*Fgf4*).

### 2.5. Changes in the TIB Muscle Transcriptome Related to Intermediary and Energy Metabolism

**Figure 4:**
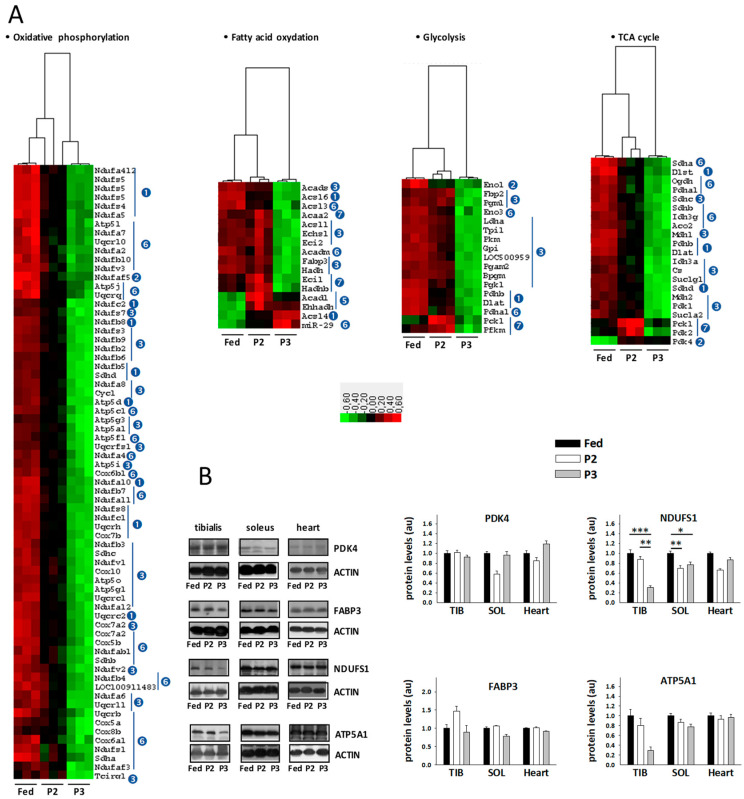
Changes related to energy/fuel metabolism in tibialis anterior muscle of rats during prolonged fasting. (**A**) Heatmaps showing differentially abundant transcripts between fed, phase 2 (P2), and phase 3 (P3) animals (*N* = 3/group). Red, black, and green boxes represent downregulated, intermediate, and upregulated genes, respectively. Legend for numbers in blue circles, which indicate when mRNA levels were significantly changed (ANOVA and Tukey *p*-values < 0.001) is given in Figure 2B. (**B**) Representative Western blots and densitometry data for muscle proteins (means ± SEM, *N* = 5/group). The values in fasted animals were normalized to those in fed rats that were assigned an arbitrary value of one. * *p*-value < 0.05, ** *p*-value < 0.01, *** *p*-value < 0.001 (one-way ANOVA and Tukey tests). PDK4: pyruvate dehydrogenase kinase isoform 4; FABP3: fatty acid-binding protein; NDUFS1: nicotinamide adenine dinucleotide[NADH]-ubiquinone oxidoreductase 75 kDa subunit; ATP5A1: ATP synthase subunit alpha; au: arbitrary units.

Numerous changes were observed for transcripts coding factors essential to fuel and energy metabolism (Figure 4A, see also details in Table S1). Compared to the fed state, P2 of fasting was notably characterized by the reduced expression of several subunits from the mitochondrial oxidative phosphorylation (OXPHOS) complexes, especially complex I (nicotinamide adenine dinucleotide[NADH] dehydrogenase, 18 transcripts) but also complex II (succinate dehydrogenase, 1 transcript), complex III (cytochrome bc1 complex, 2 transcripts), complex IV (cytochrome c oxidase, 1 transcript), and complex V (ATP synthase, 1 transcript). During P3 of fasting, most of these changes were more pronounced than in P2, and downregulation became general for all subunits of OXPHOS complexes in P3 versus the fed state. Hence, we observed the reduced expression of additional subunits from complex I (23 transcripts), complex II (3 transcripts), complex III (6 transcripts), complex IV (8 transcripts), and complex V (10 transcripts). Concerning glycolysis, three transcripts (*Dlat*, *Eno1*, *Pdhb*) were significantly less expressed in P2 versus the fed state. These changes were maintained or exacerbated during P3 of fasting, where the additional downregulation of the abundance of 12 transcripts was observed in comparison to the fed state. For the pentose phosphate pathway, upregulation of hexose-6-phosphate dehydrogenase (*H6pd*) was observed in both P2 and P3 of fasting. For fatty acid oxidation, the upregulation of three transcripts (*Acadl*, *Acsl4*, and *Ehhadh*) and downregulation of one transcript (*Acsl6*) were observed during P2 versus the fed state and changes for *Acsl2* and *Acsl6* were more marked during P3. In addition, during P3, significant downregulation of eight other transcripts (*Acadm*, *Acads*, *Acsl1*, *Acsl3*, *Echs1*, *Eci2*, *Fabp3*, *Hadh*) was observed in comparison to the levels measured in fed animals, whereas the levels of miR29a were higher. Fasting-induced downregulation was also seen at the level of the tricarboxylic acid cycle (TCA) for 4 transcripts (*Dlat*, *Sdhd*, *Dlst*, *Pdhb*) during both P2 and P3 of fasting, and for 13 transcripts (*Aco2*, *Cs*, *Idh3a*, *Idh3g*, *Mdh1*, *Mdh2*, *Ogdh*, *Pdha1*, *Sdha*, *Sdhb*, *Sdhc*, *Sucla2*, *Suclg1*) in only P3. The levels of pyruvate dehydrogenase kinase isozyme 4 (*Pdk4*) were higher in TIB from rats in both fasting P2 and P3 versus rats in the fed state. Finally, the mRNA levels of the catalytic AMP-activated protein kinase (AMPK) subunit alpha-1 (*Prkaa1*) were increased by fasting in P3, and a trend toward upregulation was observed for the non-catalytic subunit gamma-2 (*Prkag2*), whereas the catalytic AMPK subunit alpha-2 (*Prkaa2*) remained unchanged and several non-catalytic subunits beta and gamma tended to be downregulated.

### 2.6. Changes in Sleletal Muscle Protein Levels in Response to Prolonged Fasting

Using Western blot analysis, we measured reduced levels for NADH-ubiquinone oxidoreductase 75 kDa subunit (NDUFS1) during P2 versus the fed state in TIB and during both P2 and P3 in SOL (Figure 4B). However, protein levels remain unchanged for pyruvate dehydrogenase kinase isozyme 4 (PDK4), fatty acid-binding protein (FABP3), and ATP synthase subunit alpha (ATP5A1). We also observed lower levels for endoplasmic reticulum (ER) chaperone BiP (HSPA5 or GRP78) in the heart from P2 and P3 versus fed rats, and higher levels for catalase in TIB and SOL from P2 and especially P3 versus fed animals (see below).

It should be noted that unchanged protein levels for TIB PDK4, FABP3, and ATP5A1 were paralleled by the significant alteration of their mRNA levels. Moreover, the decrease in TIB *Ndufs1* mRNA levels especially pronounced in P3 was paralleled by a decrease in TIB NDUFS1 protein levels significant only in P2. In rat TIB, changes in CAT protein levels were in line with changes in *Cat* mRNA levels observed using Northern blots. The same is true in rat SOL and heart, except from the fact that significance was not systematically reached in all cases. Finally, concerning HSPA5, it remained unchanged both at the protein (Western blot) and mRNA (microarray) levels.

### 2.7. Changes in Skeletal Muscle Metabolite and Cofactor Levels in Response to Prolonged Fasting

**Figure 5:**
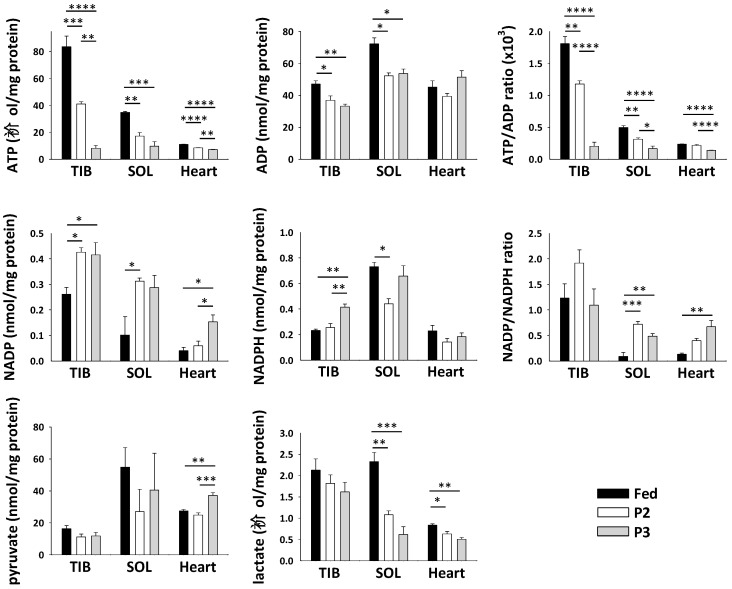
Changes in levels of metabolites and cofactors in muscles of rats during prolonged fasting. Levels of ATP, nicotinamide adenine dinucleotide (ADP), oxidized (NADP) and reduced (NADPH) forms of nicotinamide adenine dinucleotide phosphate, pyruvate, lactate, and the ATP/ADP and NADP/NADPH ratios in tibialis anterior (TIB), soleus (SOL), and heart muscles from fed, phase 2 (P2), and phase 3 (P3) rats are presented as the means ± SEM (*N* = 5/group). * *p*-value < 0.05, ** *p*-value < 0.01, *** *p*-value < 0.001, **** *p*-value < 0.0001 (one-way ANOVA and Tukey tests).

To further evaluate the functioning of TIB, SOL, and cardiac muscles during prolonged fasting, we measured the levels of various cofactors and metabolites (Figure 5). The levels of ATP were significantly decreased by both P2 and P3 in all three muscles, as well as those of adenosine diphosphate (ADP) in TIB and SOL. The ATP/ADP ratio was lower due to fasting in all three muscles. On the reverse, the levels were higher during P2 and P3 of fasting versus the fed state for the oxidized form of nicotinamide adenine dinucleotide phosphate (NADP) in all three muscles, whereas the levels of its reduced form (NADPH) were increased by P3 in TIB. Consequently, the NADP/NADPH ratio was increased during fasting, especially in SOL and cardiac muscles. Finally, muscle levels of pyruvate were significantly increased only in P3 versus fed rats, and levels of lactate were decreased by fasting in SOL and heart, but significance was not reached in TIB.

### 2.8. Changes in the TIB Muscle Transcriptome Related to Oxidative Stress

**Figure 6:**
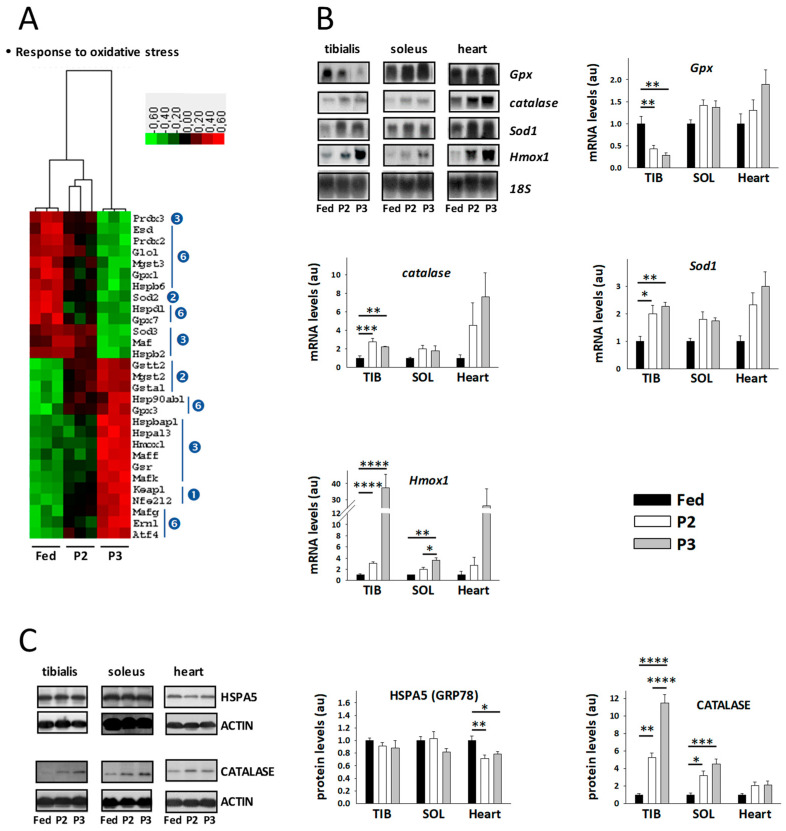
Changes related to oxidative stress in tibialis anterior muscle of rats during prolonged fasting. (**A**) Heatmaps showing differentially abundant transcripts between fed, phase 2 (P2), and phase 3 (P3) animals (*N* = 3/group). Red, black, and green boxes represent downregulated, intermediate, and upregulated genes, respectively. Legend for numbers in blue circles, which indicate when mRNA levels were significantly changed (ANOVA and Tukey *p*-values < 0.001), is given in Figure 2B. (**B**) Representative Northern blots and densitometry data for muscle mRNAs (means ± SEM, *N* = 5/group). (**C**) Representative Western blots and densitometry data for muscle proteins (means ± SEM, *N* = 5/group). The values in fasted animals were normalized to those in fed rats that were assigned an arbitrary value of 1. * *p*-values < 0.05, ** *p*-values < 0.01, *** *p*-values < 0.001, **** *p*-values < 0.0001 (one-way ANOVA and Tukey tests). Sod1: superoxide dismutase 1; *Gpx*: glutathione peroxidase; *Cat*: catalase; *Hmox1*: heme oxygenase 1; HSPA5 (GRP78): ER chaperone BiP; au: arbitrary units.

Transcriptomic changes for the factors known to be involved in the response to oxidative stress are illustrated in Figure 6A,B. In comparison to the fed state, only the levels of mitochondrial superoxide dismutase (*Sod2*) were found at significantly lower levels during P2 of fasting, whereas at the same time transcript levels for factors of the glutathione system (*Gsta1*, *Gstt2*, *Mgst2*) and for Kelch-like ECH-associated protein 1 (*Keap1*) and the transcription factor NRF2 (*Nfe2l2*) were upregulated. These changes were all maintained during P3 of fasting, and the levels of *Keap1* and *Nfe2l2* were even significantly more markedly increased. Additional changes occurred during P3 versus the fed state. Lower levels were indeed recorded in P3 relative to fed rats for transcripts coding factors of the glutathione system (*Esd*, *Gpx1*, *Gpx7*, *Glo1*, *Mgst3*), antioxidant enzymes (*Sod3*, *Prdx2*, *Prdx3*), heat shock proteins (*Hspb2*, *Hspb6*, *Hspd1*), and the transcription factor Maf (*Maf*). On the reverse, higher levels were observed in P3 relative to fed rats for transcripts coding factors of the same systems, i.e., the glutathione system (*Gpx3*, *Gsr*), one antioxidant enzyme (*Hmox1*), heat shock proteins (*Hspa13*, *Hspb90ab1*, *Hspbap1*), transcription factors involved in the action of NRF2 (*Maff*, *Mafg*, *Mafk*), and factors involved in ER stress (*Atf4*, *Atf6*, *Ern1*). It is of note that transcripts for catalase (*Cat*), glutathione S-transferase mu 2 (*Gstm2*), and superoxide dismutase [Cu-Zn] (*Sod1*) were not significantly regulated by fasting using the adjusted *p*-value threshold of *p* < 0.001, but an upregulation was observed for Cat and Gstm2 (1.5–3.3 times) during fasting.

Using Northern blot analysis (Figure 6B), fasting-induced upregulation of the levels of *Hmox1*, *Sod1*, and *Cat* was confirmed in TIB, which was extended to SOL and heart muscle, although it was not systematically significant. We also observed decreased levels of *Gpx1* in TIB, but no significant change in the two other muscles.

### 2.9. Changes in the TIB Muscle Transcriptome Related to Muscle Secretory Function

Concerning gene expression of myokines, we could observe significantly higher levels of interleukin 15 (*Il15*) in P2 compared to the fed state, and of osteocrin (*Ostn*) in P3. Conversely, gene expression of fibronectin type III domain-containing protein 5 (*Fndc5*) was significantly lower in both P2 and P3 rats versus fed animals. The other myokine mRNAs we measured (interleukin-6, *Il6*; brain-derived neurotrophic factor, *Bdnf*; myostatin, *Mstn*, fibroblast growth factors 19 and 21, *Fgf19* and *Fgf21*; decorin, *Dcn*; apelin, *Apln*; osteonectin, *Sparc*; erythroferrone, *Fam132b*) did not display significant changes.

## 3. Discussion

**Figure 7:**
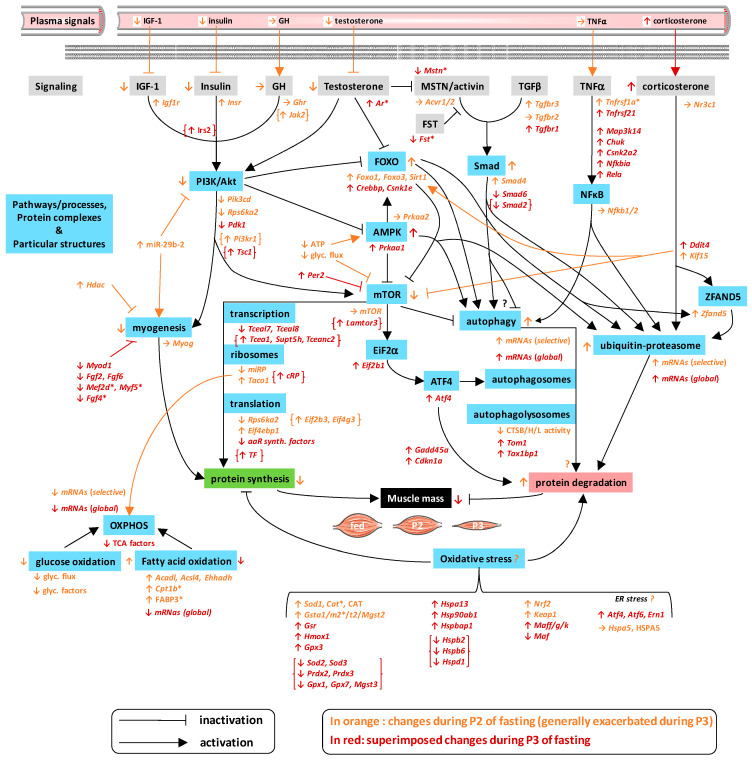
Molecular regulations controlling muscle mass during prolonged fasting in rats. This figure summarizes the molecular changes we observed in rat skeletal muscle during P2 and P3 of prolonged fasting compared to fed state. Significant changes in mRNA (ANOVA and Tukey *p*-values < 0.001) and protein abundances (ANOVA and Tukey *p*-values < 0.05) are shown in italic and capital letters, respectively. Non-significant changes but exhibiting a clear trend are highlighted with a “*”. The vast majority of muscle regulations were in line with downregulation of protein synthesis and upregulation of protein degradation during P2 and more markedly P3 of fasting. Opposite regulations are shown between braces. Glyc. flux: glycolytic flux; aaR synth.: aminoacyl-transfer ribonucleic acid (tRNA) synthesis; miRP: mitochondrial ribosomal proteins; cRP: cytosolic ribosomal proteins; TF: translation factors.

Because muscle tissue is essential for body homeostasis and functioning [1,2,3,4], and it contains a large part of the body’s proteins, being necessary to unveil how the mechanisms that control its mass are regulated. This is particularly true in the case of wasting conditions, for which there is no effective treatment to prevent muscle atrophy and associated metabolic disorders. Short-term food deprivation has already been used as a model to study the transcriptional changes that underlie the loss of body proteins, including muscle proteins. However, it is widely recognized that body proteins are progressively mobilized at the lowest rates during fasting on the short/medium term. Instead of using amino acids to produce glucose through gluconeogenesis, production of ketone bodies and intense mobilization of fat stores help spare glucose as well as body proteins. By contrast, long-term food deprivation has still been poorly studied, although many wild animals reach this stage during specific activities, such as migration or breeding. In particular, the study of the specific changes that occur during the successive metabolic phases of body protein sparing (P2, short/medium term) then increased utilization in body protein (P3, long term) has been neglected until now [13,16,17,18,19,20]. To our knowledge, only the targeted measurement of few mRNA levels [19,20] but no omics analysis has been performed previously in muscles during prolonged fasting. To fill this gap, we performed an in-depth investigation of muscle transcriptome from the fed state to the successive metabolic phases of fasting. The first striking observation we made was that the overall transcriptomic response of skeletal muscle to prolonged fasting reported here appears somewhat peculiar compared to those of the liver [32] and adipose tissue [33]. Indeed, skeletal muscle mRNA expression profiles in P2 rats were closer to those in fed rather than those in P3 animals (Figure S1), whereas a clear distinction was seen between fed and fasted (both P2 and P3) states for hepatic and adipose gene expressions [32,33]. This is consistent with the significant loss of mass of the liver and adipose tissue, which occurs already in P2 compared to fed state [32,33], whereas it can be seen here for TIB that the loss of mass became significant only in P3 animals. During P2 of fasting, the induction of protein sparing mechanisms, as reflected by low plasma urea levels, no doubt contributes to the preservation of muscle mass and function. Protein sparing during P2 has notably been linked to lipid fuel availability [34,35,36]. The shift toward ketone body production and the preferential utilization of fatty acids and ketone bodies at this stage likely help spare amino acids and glucose [16]. Moreover, lipids can themselves interact with signaling pathways that control muscle mass [37,38]. We thereafter discuss in detail the molecular regulations we observed in muscles in response to prolonged fasting, the main regulations being related to the control of protein balance and energy metabolism (Figure 7 and Figure S1).

### 3.1. Changes Related to Muscle Protein Degradation

Muscle protein degradation is controlled by numerous hormones, such as steroid hormones and cytokines [24,25,26,27]. TGFβ is known to inhibit myogenesis; it also activates the Smad pathway and has been shown to possibly trigger muscle atrophy [39]. TGFβ can also act via a Smad-independent catabolic pathway, involving TRAF6-mediated activation of NFκB and MAP kinases [40]. In addition, myostatin (MSTN), a TGFβ family member, is known to inhibit protein synthesis via the AKT pathway and to promote proteolysis through the Smad pathway [41,42]. Finally, activin is another TGFβ family member working in concert with MSTN, and it is also able to activate Smad proteins while its action is counteracted by follistatin [43]. Changes in mRNA levels for the factors involved in TGFβ signaling (*Tgfbr1*, *Tgfbr3*, *Smad4*, and *Smad6*) may play a role in TGFβ–Smad-mediated protein degradation during prolonged fasting. On the reverse, except from the upregulation of *Map2k3* mRNA levels during P3, transcriptional regulation does not seem to be involved in the possible role of the Smad-independent pathway mediated by TRAF6. Because the TRAF6 pathway is known to notably upregulate expression of collagen alpha-1(I) chain (*Col1a1*) in various tissues, the clear drop in *Col1a1* mRNA levels that we observed here, although it was not significant due to interindividual variations during fasting (Table S1), may indicate that this pathway is not involved in muscle protein wasting during prolonged fasting. During P3, we also observed a trend for decreased follistatin (*Fst*) mRNA levels, which may support alleviation of its inhibitory effect on MSTN/activin signaling. Hence, these few transcriptional regulations highlight specific molecular responses that are in line with previous results showing that muscle proteolysis is increased essentially during P3 [21]. During P3, the opposite is expected from downregulation of *Smad2*, and to a lesser extent from the trend to decrease of *Acvr1* and *Smad3* gene expressions.

TNFα is a well-known proteolytic factor acting through NFκB [44]. Maintenance of its circulating levels during prolonged fasting, as well as increased gene expression of *Csnk2a2* already from P2 of fasting but more markedly during P3, and the significantly increased gene expression of *Map3k14*, *Chuck*, *Rela*, and *Nfkbia* during P3 only (Figure 2) support an activation of muscle NFκB signaling in late fasting. However, because NFκB can stimulate autophagy or activate autophagy inhibitors [45], it is difficult to predict how NFκB signaling influences autophagy during long-term food deprivation.

Corticosterone is the main steroid hormone that is involved in protein degradation [46]. Its plasma levels and muscle gene expression for *Ddit4* were significantly elevated only during P3, despite a clear trend already in P2. Moreover, *Klf15* mRNA levels were increased in P2 and more markedly in P3. During P3 of fasting in particular, such regulations appeared not only to be in favor of protein catabolism, but also against protein synthesis. Indeed, KLF15 is a transcription factor regulating transcription of *Foxo1*, *Fbox32*, and *Trim63*, and both KLF15 and DDIT4 inhibit mTOR activity [46].

Most of the signaling pathways that control muscle protein balance converge to the FOXO pathway [47]. At the mRNA level (Figure 2), upregulation of *Foxo1*, *Foxo3*, and *Sirt1* in P2 and more markedly in P3, as well as the additional upregulation of *Crebbp* and *Csnk1e* during P3, likely reflect an activation of the FOXO pathway, which is expected to positively influence downstream factors during fasting. FOXO notably controls muscle protein degradation through regulation of the autophagy and ubiquitin–proteasome systems [48,49]. By examining gene expression of few proteolytic factors only, we have already reported elsewhere that induction of proteolytic systems in skeletal muscle appear selective during P2 then coordinated during P3 [19]. Data from the current study validate this hypothesis on a global transcriptomic scale (Figure 2). Consistent with the protection from starvation-induced muscle atrophy that is observed when the proteasome is inhibited [50], partial induction only of proteasome subunit gene expression during P2 of fasting may help limit protein breakdown, i.e., favor protein sparing, whereas an overall induction during P3 no doubt favors muscle proteasomal proteolysis. In situations such as short-term fasting, denervation, or high doses of glucocorticoids, a role for ZFAND5 in muscle atrophy has been reported, likely through enhancement of the proteasome activity [51]. Our findings showing an induction of *Zfand5* gene expression in P2 and more markedly in P3 animals support the importance of this catabolic pathway during prolonged fasting. Concerning autophagy, ATF4 expression has been reported as an important factor for muscle atrophy in various conditions, including fasting, through mechanisms that are not fully understood yet [52]. ATF4 synthesis is under the control of eukaryotic initiation factor 2 alpha (eIF2α) kinase activity [52]; it has notably been involved in autophagosome formation [53], and its pro-atrophic effects involve mRNA expression of, notably, *Gadd45a* and *Cdkn1a* [52]. Looking more precisely at the eIF2α–ATF4 axis in our data (Figure 2), we could see that increased mRNA levels of *Eif2b1*, *Atf4*, *Gadd45a*, and *Cdkn1a* occurred particularly during P3 of fasting. This may represent key regulations for increasing protein breakdown in late fasting. It is possible that autophagosome formation is enhanced during fasting, notably P3, as well as ATF4-mediated proteolysis. Moreover, alteration in gene expression for factors playing important roles in autophagosome maturation and fusion with the lysosome [54] was observed in the current study (Figure 2). Indeed, the mRNA levels of *Tom1* and *Tax1bp1* were higher during P3 of fasting, but those of *Optn* and *Myh6* remained unchanged. On the whole, autophagy therefore appears to be induced during prolonged fasting. Accordingly, the activity of cathepsin D is increased during short-term fasting [55]. It is of note that the activity of three other muscle cathepsins has been shown to be decreased during P2 and P3 of fasting [23], which may indicate additional alteration of the autophagy process that would require further investigation.

### 3.2. Changes Related to Muscle Protein Synthesis

Multiple pathways control protein synthesis and muscle growth [24,25,26,27]. During P2 of fasting, and more markedly during P3, increased gene expression for insulin and IGF-1 receptors (*Insr* and *Igf1r*) may represent regulatory responses to decreased levels of circulating insulin (Figure 1) and IGF-1 [56]. Moreover, the induction of GH signaling might not be affected during fasting, as reflected in maintained levels of circulating GH and muscle *Ghr* gene expression (Figure 1 and Figure 3). IGF-1 notably increases myogenesis, and its decreased plasma levels may therefore support repression of myogenesis during fasting. Numerous other factors are key to muscle differentiation and regeneration processes [57,58], and the levels of several of them were altered during prolonged fasting in rat muscles (Figure 3). In particular, increased abundance of miR-29b-2 was the only change that could be in favor of muscle cell differentiation in both P2 and P3 rats. On the reverse, this could also contribute to muscle wasting during prolonged fasting since miR-29b overexpression is sufficient to promote muscle atrophy in vivo, notably through interaction with IGF-1 signaling [59]. Although *Myog* expression remained unchanged, increased *Hdac4* mRNA levels in P2 and P3, as well as downregulation during P3 of *Myod1*, *Fgf2*, and *Fgf6* gene expressions, and the trends for decreased abundance of *Fgf4*, *Mef2d*, and *Myf5* in P3, all appear as molecular events that do not favor muscle development and regeneration during prolonged fasting.

Like the vast majority of peripheral organs, the skeletal muscle harbors a circadian clock, which modulates various aspects of muscle physiology, such as muscle mass and strength [60,61]. Among clock genes, gene expression of *Per1* and *Per2* was upregulated by fasting during P2 and more markedly during P3, whereas mRNA levels of *Bhlhe40* or *Dec1* were decreased (Figure 3). It is possible that *Dec1* downregulation is linked to *Per* upregulation because PER (and cryptochrome (CRY)) proteins reduce transcription of *Dec1* [62]. However, because regulatory loops within the circadian clockwork are interlocked, this interpretation would need to be tested by further investigation. Nevertheless, the present data on *Per* and *Dec1* in rat muscle clearly confirm earlier findings in other peripheral organs (e.g., heart, liver, and intestine) of fasted mice [63], although that study did not take into account P2 and P3 stages. Overexpression of DEC1 has been reported to inhibit myogenesis by decreasing gene expression of muscle-specific transcription factors such as *Myf5*, *MyoD*, and *MyoG* [64]. It remains difficult to know if decreased *Dec1* mRNA levels during prolonged fasting are accompanied by an increase in its translation, or if they simply rule out the possible involvement of DEC1 in fasting-induced changes for *Myod1* and *Myf5* (see above). The mTOR pathway is closely interacts with the clockwork [65]. Of note, increased expression of PER2 has been reported to suppress the activity of the mTOR complex 1 (mTORC1) in the cytosol of hepatocytes [66]. Similar changes that lead to reduced protein synthesis in the fasted liver may thus occur in the muscle during P2 and further during P3. On the whole, alteration of clock-related gene expression may indicate circadian changes that could be specific to the phases of fasting. Therefore, further investigations taking into account transcriptional changes according to times of day and phases of prolonged fasting will be needed to refine the understanding of food deprivation-induced responses.

Testosterone is a steroid hormone with anabolic effects on muscle mass and strength, which notably involves positive effects on myogenic differentiation, as well as crosstalk with other signaling molecules such as AKT, mTOR, FOXO, myostatin, and IGF-1 [67]. Therefore, downregulation of testosterone circulating concentrations during both P2 and P3 of prolonged fasting (Figure 1) may support repression of myogenesis and muscle protein synthesis during prolonged fasting, while favoring muscle protein breakdown.

Concerning downstream signaling pathways for insulin, IGF-1, and GH receptors, the gradual decrease in muscle protein synthesis rate during prolonged fasting [13] appears supported by downregulation of the levels of *Pik3cd* and *Rps6ka2* mRNA and by upregulation of *Eif4ebp1* mRNA levels during P2. The same applies to the additional decrease in expression levels of *Pdk1*, the two transcription elongation factors *Tceal7* and *Tceal8*, and numerous genes involved in aminoacyl-tRNA synthesis during P3 (Figure 3). For example, the *Rps6ka2* gene encodes a member of the p90 ribosomal S6 kinase (RSK) family of serine/threonine kinases, some of which have been reported to promote mTOR signaling through phosphorylation of tuberin [68]. In addition, lack of PDK1 during P3 could worsen the situation, as many proteins involved in protein synthesis and cell growth regulation are known to be phosphorylated and activated by PDK1, including RSK proteins, p70 ribosomal S6 kinases (S6K), and many protein kinase C (PKC)/AKT isoforms [69]. At the mitochondrial level, specific ribosomes operate translation for 13 proteins, all of them subunits of the mitochondrial membrane respiratory chain [70]. We observed a global downregulation for gene expression of mitochondrial ribosomal proteins during fasting, especially during P3. Moreover, TACO1 is the only mitochondrial translational activator known to date [70], and its gene expression was also reduced by fasting in rats in P2 and P3. Hence, these regulations likely reflect the reduction in the amount of OXPHOS complexes and in metabolic rates during prolonged fasting. Because there is increasing evidence that mitochondrial translation is adapted to cytoplasmic protein synthesis [71], the regulations we describe here for mitochondria might indicate decreased levels of also nuclear-encoded OXPHOS subunits. In the end, these different mechanisms bring together a number of regulations at the transcriptional level, which likely favor downregulation of muscle protein synthesis during P2 but more markedly during P3 of fasting.

We also observed an induction for numerous gene expressions, including those for *Jak2*, *Pi3kr1*, *Eif2b3*, and *Eif4g3* during P2, to which the upregulation of *Irs2*, *Tsc1*, and *Lamtor3*; the transcription elongation factors *Tcea1*, *Supt5h*, and *Tceanc2*; and many cytosolic ribosomal proteins and translation factors during P3 are added (Figure 3). Such regulations may help maintain protein turnover at a level compatible with cell functioning, by limiting the reduction in protein synthesis. This may represent mechanisms contributing to muscle sparing, especially during P2. During P3, we could hypothesize an anticipation of food intake for replenishment of the protein compartment to be prioritized in case of refeeding. Wild animals undergoing prolonged fast to breed are indeed abandoning their duty for refeeding when reaching P3 [72]. Moreover, an anticipation has already been reported in rats submitted to prolonged fasting, with a restoration of the intestine before food is yet available [73]. An alternative explanation could involve the increase in locomotor activity in P3 fasting rats [74,75]. Because exercise is known to result in changes in gene transcription that ultimately improve muscle performance through notably regulation of protein synthesis [76], it is tempting to propose that the increased drive for refeeding in late fasting (P3) triggers an increase in locomotor activity, which in turn induces transcriptional regulations in favor of protein synthesis.

### 3.3. Changes Related to Muscle Energy Metabolism

At the level of the whole organism, prolonged fasting is characterized by the successive use of different sources of energy, with carbohydrate stores being exhausted after a short period of adaptation (phase 1), lipids being the prominent fuel during P2, and a rise in protein utilization being observed during P3 [16,17,18]. This is nicely reflected in the RQ values that we obtained (Table 1).

At the muscle level, transcriptional regulation (Figure 4) highlights a global decrease for glycolytic factors during P2 and more markedly during P3, which accords with the expected decreased use of carbohydrate fuels during prolonged fasting. Low-glucose conditions have been shown to inhibit mTOR signaling in vitro [77]. Circulating glucose concentrations were decreased during prolonged fasting (Table 1), as well as muscle lactate levels (Figure 5). Therefore, it could be that low glycolytic flux in prolonged fasted rats contributes to muscle wasting through suppression of mTOR-induced protein synthesis.

Concerning fatty acid oxidation, among the very few changes that were observed during P2, increased mRNA levels of *Acadl*, *Acsl4*, and *Ehhadh*, as well as the trend for increased carnitine O-palmitoyltransferase 1 (*Cpt1b*) mRNA and FABP3 protein levels likely support the preferential use of lipid fuels at this stage (this is the reverse for decreased *Acsl6*). Then, although this is not the case for *Acsl4*, mRNA levels of *Acadl* and *Ehhadh* were lower during P3 compared to P2 and all other fatty acid oxidation factors were downregulated compared to the fed state, which supports the reduced contribution of lipid fuels to energy expenditure in late fasting. Moreover, transcriptional regulation at the level of TCA also supported the reduced oxidation of lipid fuels and glycolytic intermediates, since changes were restricted to a minimum during P2 of fasting, essentially having trends toward downregulation. Downregulation then became generally significant during P3. miRNA-29a is highly expressed under insulin-resistant conditions and it plays a key role in alteration of genes associated with lipid metabolism [78]. These may be the reasons why we observed higher levels during P3.

The pentose phosphate pathway generates NADPH, a reducing equivalent necessary for anabolic reactions [79]. Unchanged mRNA levels for glucose-6-phosphate 1-dehydrogenase (*G6pdx*) and 6-phosphogluconolactonase (*Pgls*) suggest NADPH production is not altered in the cytosol. Accordingly, assaying NADPH in the whole muscle revealed unchanged levels but a trend toward an increase during P3 of fasting in TIB but not in the two other muscles (Figure 5). NADPH therefore does not seem to be a limiting factor for macromolecule synthesis during food deprivation. Oxidized NADP is known to affect calcium homeostasis by favoring mobilization of calcium stores [79]. Increased NADP levels in all three muscles during prolonged fasting therefore no doubt help muscle function to be sustained, but the exact mechanisms remain to be determined.

Together with the decreased abundance of NDUFS1 at the protein level during P3 of fasting, the general downregulation of gene expression for OXPHOS proteins, also especially pronounced during P3 (Figure 4), is in perfect accordance with decreased metabolic rates (Table 1) and muscle ATP levels (Figure 5). In humans, whole-body metabolic rate and substrate oxidation appear to be largely determined by enzymatic activities in skeletal muscle [80]. Our data suggest that transcriptional regulation of muscle fuel/energy metabolism is also a key determinant of whole-body energy consumption, at least during fasting. The fact that NDUFS1 protein levels were not significantly changed in the heart could reflect the expected preservation of cardiac activity during fasting.

AMP-activated protein kinase (AMPK) is central in controlling cellular energy homeostasis. It has been shown to be a prime sensor of the lower availability of glucose during fasting, also triggering the adapted response to favor lipid utilization [81]. AMPK is also sensitive to low energy conditions, being activated when ATP concentrations and the ATP/ADP ratio are low [82], which was the case in prolonged fasted rats for all three muscles studied in P3 (Figure 5). AMPK generally activates catabolic factors while inactivating anabolic ones, thereby controlling energy metabolism and muscle growth or atrophy [83]. The regulation of muscle mass notably appears to be controlled by the prominent role of AMPK subunit alpha-1 in stimulating anabolism, while AMPK subunit alpha-2 seems to play a more important role in the control of muscle catabolism during atrophy. Increased mRNA levels of AMPK subunit alpha-1 (*Prkaa1*) during P3 of fasting therefore accord with a possible important role of AMPK in limiting the drop in protein synthesis, while unchanged levels for *Prkaa2* could favor protein degradation. AMPK is notably inhibiting protein synthesis through repression of mTOR signaling and inhibition of eukaryotic elongation factor 2 (*eEF2*) activity, and it is known to promote muscle autophagy as well as ubiquitin–proteasome mediated proteolysis, notably through stimulation of FOXO activity [83]. Hence, transcriptional regulation of AMPK reflects its central role as a controller of muscle metabolism and protein balance during prolonged fasting, acting to inhibit protein synthesis while favoring protein breakdown and limiting ATP depletion.

### 3.4. Changes Related to Muscle Response to Oxidative Stress

Importantly in catabolic situations, oxidative stress has been connected to promotion of muscle proteolysis and inhibition of protein synthesis, thereby contributing to muscle atrophy [84]. Oxidative stress occurs when the production of oxidant compounds such as reactive oxygen species (ROS) exceeds the protection provided by antioxidant systems (PRDXs) [85]. During prolonged fasting, oxidative stress has been shown to be exacerbated in the liver of P3 rats [32]. Increased ROS levels have also been shown to be, at least partly, responsible for fasting-induced skeletal muscle atrophy in food-deprived C2C12 myotubes [86].

Mitochondria are an important source of ROS within most mammalian cells, notably at the level of the respiratory chain [87]. The regulations we saw here in terms of mRNA and protein abundance changes (see above) might contribute to the impairment of activity of the mitochondrial respiratory chain during prolonged fasting, and superimposition of reduced fuel oxidation (see above) may contribute to suppress electron supply to the respiratory chain. Although we cannot definitely conclude, such data could indicate a reduction of mitochondrial ROS production in rat skeletal muscle during prolonged fasting. To determine if prolonged fasting triggers muscle oxidative stress, it is unfortunate that we did not collect enough samples to assay oxidative damages in muscles from prolonged fasted rats. This should obviously be measured in future studies.

Looking at regulations for antioxidant defenses may give indices. We obtained results suggesting that oxidative stress occurs in rat muscle during prolonged fasting, but data were somewhat contrasted (Figure 6). Indeed, decreased levels of *Sod2* and *Sod3* mRNAs but increased levels of *Sod1* mRNA levels during P2 and especially P3 of fasting may reflect the fact that there is no need for increasing dismutation of superoxide anion (O_2_•−) to form hydrogen peroxide (H_2_O_2_) in the mitochondria and extracellular space, but cytosolic detoxification may be required. Then, increased levels of catalase (mRNA and protein) in both P2 and P3 of fasting could reflect the need for reduction of H_2_O_2_ to water, and they may help in detoxification. It should be noted here that *Cat* and *Sod1* mRNAs measured in rat TIB did not exhibit perfectly matched changes when comparing microarray and Northern blot analyses. Lack of significance in microarray analysis could be due to the small sample size, as well as the use of very low *p*-value thresholds. Alternatively, discrepancy could come from different sensitivity of the techniques. It is more difficult to interpret the lower levels of *Prdx2* and *Prdx3* mRNAs during P3. ROS scavenging and reduction of lipid hydroperoxides by the glutathione system is particularly important to protect cells against oxidative stress [88]. In that sense, upregulation of *Gpx3* gene expression, especially during P3 of fasting, likely reflects increased peroxidation levels in the extracellular region at this stage, whereas decreased gene expression for *Gpx1* and *Gpx7* may reflect the fact that catalase is sufficient to detoxify H_2_O_2_ within cells. Apart from the drop in *Mgst3* mRNA levels, upregulation of a number of other glutathione S-transferases (GSTs) and of glutathione reductase (*Gsr*) is also reflective of oxidative stress due to lipid hydroperoxides during P2 and more markedly during P3 of fasting. Apart from *Gpx1* mRNA levels, the assays we performed in oxidative muscles (SOL and heart) were globally in line with what we found in TIB, thus suggesting an equal impact of fasting-induced oxidative stress in all muscles.

NADPH is one of the most important factors in cellular response to oxidative stress, notably as a cofactor for glutathione reductase [79]. The trend for increased NADPH levels, at least in TIB, may therefore help fight oxidative stress during prolonged fasting. Heat shock proteins are not only molecular chaperones, but also they help fight oxidative stress [89]. The induction of *Hspa13*, *Hsp90ab1*, and *Hspbap1* in skeletal muscle of P3 rats may therefore be involved in response to oxidative stress during prolonged fasting, whereas the reverse could be interpreted from decreased levels of *Hspb2*, *Hspb6*, and *Hspd1* mRNA levels.

Nuclear factor, erythroid 2-like 2 (NFE2L2 or NRF2) regulates antioxidant enzyme expression [90]. Its increased gene expression (Figure 6), together with increased mRNA levels for its coworkers, *Keap1*, and the small Maf proteins (*Maff*, *Mafg*, and *Mafk*), as well as downregulation of the inhibitory musculoaponeurotic fibrosarcoma oncogene (cMaf) transcription factor (*Maf*) [91], argue for a role of NRF2 in inducing gene expression of antioxidant enzymes during P2 and especially P3 of fasting, including *Hmox1*, *Sod1*, *catalase*, *Gpx3*, and most of *Gsts*. Interestingly, phosphorylation is not the only activating pathway for NRF2, and a role for ATF4 has been recently reported [92]. ATF4, ATF6, ERN1, and HSPA5 are major factors in the response to ER stress [93]. The higher levels of *Atf4*, *Atf6*, and *Ern1* mRNAs during P3 and the maintenance of HSPA5 mRNA and protein levels may be reflective of ER stress. Therefore, upregulation of *Atf4* mRNA levels could constitute a regulation to favor NRF2 protective response after ER stress-induced ROS production during P3 of fasting.

### 3.5. Changes Related to Muscle Secretory Function

Skeletal muscle actively secretes a range of proteins collectively referred to as myokines [9]. Several of these myokines exert paracrine or autocrine effects and play a role in the regulation of skeletal muscle mass and function [94,95]. Gene expression of musclin, which is also termed osteocrin (OSTN), has already been shown to be drastically decreased in gastrocnemius muscle from 48h-fasted mice [96]. We show here that such decrease is also found in rat TIB, especially during P3. It has also been reported previously that mRNA levels of brain-derived neurotrophic factor (BDNF) are higher in the vastus lateralis muscle from 48 h-fasted humans [97], however BDNF gene expression remained unchanged in rat TIB in our study. Discrepancies depending on the species and/or muscle considered is not surprising. In 24 h-fasted mice, gene expression of fibronectin type III domain-containing protein 5 (FNDC5) and insulin-like growth factor 1 (IGF-1) have been shown to be reduced in SOL as well as TIB muscle from both fasted males and females [98]. We confirm here that *Fndc5* mRNA levels are also decreased in rat TIB in both P2 and P3 of fasting. In the same study [98], however, it was shown that interleukin 6 (IL6) and *Mstn* mRNA levels remained unchanged in SOL but were higher in TIB from fasted males while being lower in TIB from fasted females. In addition, myonectin (ERFE) *Fam132b* mRNA levels were higher in fasted male SOL and TIB, while being lower in fasted female TIB [98]. Finally, gene expression of fibroblast growth factor 15 (FGF19) was shown to decrease in TIB from both fasted males and females, whereas SOL expression was reduced only in males [98]. Our results showing unchanged *Mstn*, *Il6*, *Fam132b*, and *Fgf19* mRNA levels in prolonged fasted male rats therefore highlight clear differences between rats and mice. OSTN and interleukin 15 (IL15) can control muscle mass and oxidative capacity, and FNDC5 is the precursor for irisin, a myokine with a variety of metabolic effects [96]. Increased *Il15* mRNA levels in P2, and decreased *Ostn* (P3) and *Fndc5* (P2 and P3) mRNA levels (current study) are therefore expected to reflect the importance these myokines may have in response to nutritional challenges to mediate signals to the skeletal muscle itself or to remote organs.

## 4. Materials and Methods

### 4.1. Ethical Statement

The study was conducted in strict accordance with the recommendations stated in the Public Health Service policy on Human Care and Use of Laboratory Animals. All experiments were performed in conformity with the rules of the European Committee Council Directive of November 24, 1986 (86/609/EEC) and the French Department of Agriculture (license no. 67-226 to T.R.).

### 4.2. Animals and Study Design

Fifteen male Sprague Dawley rats, aged 10 weeks, were purchased from Janvier CERJ (Le Genest-St-lsle, France). They were kept in individual cages at constant ambient temperature (25 ± 1 °C) and they were exposed to a constant photoperiod (12:12, light/dark; lights on at 07:00 a.m.). During at least 1 week of acclimatization, they were fed a standard diet (UAR A04, Villemoisson, France; 50% carbohydrate, 5% fat, and 24% protein, in mass percentage), which is recommended for rearing laboratory rodents and meets National Research Council requirements, as certified by the manufacturer. When reaching approximately 345 g, the rats were subjected to the prolonged fasting protocol. For all animals, drinking water remained available ad libitum during the whole duration of the experiment.

Using a random distribution of rats, three experimental groups were formed (*N* = 5/group). Five rats were sacrificed as control-fed animals. In the post-absorptive state, they had a full stomach at the time of sampling. Five other rats were subjected to fasting until P2 and the five remaining until P3. P2 and P3 of prolonged fasting could be determined using daily weighing of rats and calculation of the rate of body mass loss (dm/m.dt), which reflects changes in the rate of protein utilization. As already known [13,19,20,31], the sparing of body proteins during P2 resulted in a slow and regular loss in body mass, whereas the increased body protein mobilization during P3 induced an accelerated loss of body mass. The metabolic status of rats was validated a posteriori by plasma metabolite and hormone measurements (*N* = 5/group). To avoid any risk that an excess of body protein loss during P3 of fasting become critical to animal survival, we sacrificed P3 rats the day after the onset of this phase. In rats with an initial body mass of approximately 400 g, rapid reversibility of 3 days of P3 had previously been shown after restoration of food availability [31]. All animals were sacrificed by cervical dislocation between 10:00 a.m. and 12:00 p.m., and truncal blood was immediately collected in tubes containing ethylenediaminetetraacetic acid (EDTA). Plasma was then prepared after centrifugation (3000× *g*; 15 min, 4 °C) and kept frozen at −80 °C until analysis. The cardiac muscle, as well as SOL and TIB, two skeletal muscles from the hindlimb, were rapidly collected and weighed. Muscle pieces were quickly frozen in liquid nitrogen and stored at −80 °C until analysis.

### 4.3. Indirect Calorimetry Measurements

Measurement of O_2_ consumption (VO_2_) and CO_2_ production (VCO_2_) was performed using an open-circuit indirect calorimetry system (Klogor, Lannion, France). Concentrations of O_2_ and CO_2_ in the outgoing air were successively measured in the different cages, one of them left vacant for determination of reference values for the ambient air. Between each measurement, 90 s was necessary to rinse the system. Final gas values were the mean of 10 measures obtained for 40 s. Each cage was sampled every 10 min, and gas analyzers were calibrated daily. Daily O_2_ consumption was the mean of all the values obtained over 24 h. The respiratory quotient (RQ) was calculated as the ratio of CO_2_ production over O_2_ consumption.

### 4.4. Plasma Metabolite and Hormone Assays

Several metabolites and hormones were assayed from plasma of fed and fasted rats (*N* = 5/group). The levels of plasma urea and glucose were measured using kits from Sigma Diagnostics (St. Louis, MO, USA) and the levels of plasma NEFA using a kit from Wako Chemicals GmbH (Neuss, Germany). Commercial ELISA kits were used to measure circulating levels of insulin (Merk Millipore, Molsheim, France), growth hormone (GH; BioVendor, Brno, Czech Republic), corticosterone (LSBio, Seattle, WA, USA), testosterone (R&D Systems, Minneapolis, MN, USA), and tumor necrosis factor alpha (TNFα; Sigma Diagnostics).

### 4.5. Muscle Metabolite and Cofactor Assays

Several metabolites and cofactors were assayed from the three muscles collected in fed and fasted rats (*N* = 5/group). The levels of lactate, pyruvate, NADP, NADPH, ATP, and ADP were measured using commercial kits from BioVision Research Products (Mountain View, CA, USA).

### 4.6. Muscle Transcriptome Analysis

Total RNA was prepared using the Trizol Reagent (ThermoFisher Scientific, Rockford, IL, USA) after frozen muscle samples (all three muscles; *N* = 5/group) had been grinded and homogenized using a laboratory ball mill. The quantity, quality, and purity of extracted RNA were assessed using a NanoDrop 1000 spectrophotometer (NanoDrop, Wilmington, DE, USA) at 260 and 280 nm and an Agilent 2100 bioanalyser (Agilent, Santa Clara, CA, USA). RNAs were considered pure for values of A260/280 ratio > 1.8 and of RNA integrity number (RIN) > 9.

DNA microarray analyses (TIB; *N* = 3/group) were carried out within the IGBMC (Institute of Genetics and Molecular and Cellular Biology, Illkirch, France) Microarray and Sequencing platform (http://www-microarrays.u-strasbg.fr/), using the GeneChip Rat Gene 1.0 ST array (Affymetrix GeneChip technology). Microarrays were scanned using an Affymetrix GeneChip Scanner 3000 7G (Affymetrix UK Ltd., High Wycombe, UK). The GeneChip Operating v1.4 (GCOS) and Expression Console v1.1 softwares (Affymetrix UK Ltd., High Wycombe, UK) allowed quantification and initial analyses. Expression levels were calculated using the following parameters: sketch quantile for normalization, median polish as in RMA for summarization. Subsequent analysis was performed on the extended set of probesets. Reference genes were eliminated. The microarray data obtained in this publication were deposited in NCBI’s Gene Expression Omnibus and are accessible through GEO Series accession number GSE149829 (https://www.ncbi.nlm.nih.gov/geo/query/acc.cgi?acc=GSE149829).

Northern blot analyses (all three muscles; *N* = 5/group) were performed following a previously described procedure [99]. Specific antisense oligonucleotide probes end-labeled (5′) with digoxigenin (Eurogentec, Seraing, Belgium) were used to target superoxide dismutase 1 (*Sod1*; 5′-CCGTCCTTTCCAGCAGCCACATTGCCCAGGTC-3′; Genbank accession number: M25157), selenium-dependent glutathione peroxidase (*Gpx1*; 5′-GGGCTCGAACCCACCACCGGGTCGGACATACT-3′; Genbank accession number: S41066), heme oxygenase 1 (*Hmox1*; 5′-GTGTGAGGACCCATCGCAGGAGCGGTGTCTGG-3′; Genbank accession number: NM_012580), and catalase (*Cat*; 5′-CCTGGAGCATCTTGTCCGGGCTGGGCTCAATG-3′; Genbank accession number: M11670). The Scion Image software v 4.02 (http://www.scioncorp.com/) allowed quantification after correction of mRNA levels for differences in gel loading or blotting by reference to the level of 18S rRNA. Quantitative values in fasted animals were normalized to those in fed rats that were assigned an arbitrary value of one.

### 4.7. Muscle Western Blot Analyses

Muscle proteins were extracted after frozen muscle samples (all three muscles; *N* = 5/group) had been grinded and homogenized using a laboratory ball mill. Addition of extraction buffer (8 M urea, 2 M thiourea, 1% Igepal, 1% dithiothreitol (DTT), Triton 0.5%, 50 µg/mL Nα-Tosyl-L-lysine chloromethyl ketone hydrochloride (TLCK), and protease inhibitors; Sigma Aldrich, St. Louis, MO, USA) to the resulting powder was followed by sonication (10 s at 135 watts) and incubation at 4 °C under agitation for 90 min. Proteins were acetone-precipitated and resuspended in Laemmli buffer. Total protein concentrations were determined using a Lowry assay (RCDC, Bio-Rad, Hercules, CA, USA).

Proteins (30 µg) were electrophoresed using 12% SDS-acrylamide gels, then transferred onto nitrocellulose membranes (Proteigene, Saint Marcel, France). After saturation overnight at 4 °C using a blocking solution (5% non-dry fat milk and 0.05% Tween-20 in tris-buffered saline (TBS), Sigma Aldrich), membranes were incubated for 3 h at room temperature with primary antibodies against ER chaperone BiP (HSPA5, diluted 1/500; ab31685 from Abcam, Paris, France), ATP synthase subunit alpha (ATP5A1, diluted 1/500; sc-58613 from Santa Cruz Biotechnology, Dallas, TX, USA), catalase (CAT, diluted 1/200; sc-34280 from Santa Cruz Biotechnology), fatty acid-binding protein (FABP3, diluted 1/200; sc-58274 from Santa Cruz Biotechnology), NADH-ubiquinone oxidoreductase 75 kDa subunit (NDUFS1, diluted 1/500; sc-50132 from Santa Cruz Biotechnology), pyruvate dehydrogenase kinase isoform 4 (PDK4, diluted 1/100; sc-14495 from Santa Cruz Biotechnology), or cytoplasmic alpha-actin (diluted 1/10000; MAB1501 from Merck Millipore, Molsheim, France). Membranes were then washed three times for 10 min (0.05% Tween-20 in TBS), then incubated for 1 h at room temperature with peroxidase-conjugated secondary antibodies, either anti-rabbit immunoglobulin G (IgG, diluted 1/5000; #7071 from Cell Signaling Technology, Boston, MA, USA), donkey anti-goat IgG (diluted 1/5000; sc-2020 from Santa Cruz Biotechnology), or anti-mouse IgG (diluted 1/5000 to 1/20000; sc-2005 from Santa Cruz Biotechnology). After three 10-min washes (0.05% Tween-20 in TBS), we added chemiluminescence reagent (ECL Western blotting substrate, ThermoScientific, Rockford, IL, USA), and hybridization signals were visualized using Amersham Hyperfilm ECL (GE Healthcare, Buckinghamshire, UK). Densitometry analysis was performed using Scion Image software v 4.02. After correction of protein levels for differences in gel loading or blotting by reference to the level of actin, we normalized quantitative values in fasted animals to those in fed rats that were assigned an arbitrary value of 1.

### 4.8. Bioinformatic Analysis

Hierarchical clustering of transcriptomics data (log_2_ mRNA abundances) was performed using the Cluster v3.0 software [100]. Only significantly changed transcripts (adjusted *p*-value below 0.001) were used. Parameters were set as follows: median centering and normalization of genes for adjusting data and centroid linkage clustering for both genes and arrays. Dendrograms were generated and visualized using the Treeview v1.1.3 program.

Enrichment and functional annotation analyses of transcriptomics data were performed using the desktop version of DAVID (Ease v2.1) and a version of GO databases downloaded in July 2019. Stringent criteria for considering significantly enriched GO terms included an Ease score lower than 0.1, a Benjamini *p*-value lower than 0.05, and a fold enrichment higher than 2. The muscle functions altered by prolonged fasting were determined after enriched GO terms had been grouped together into broad functional categories.

### 4.9. Statistical Analysis

Statistical analysis was performed using the R software environment v3.4.0 [101]. All data are presented as means ± SEM. Shapiro–Wilk (*p*-value > 0.01) and the Bartlett (*p*-value > 0.01) tests were used to check for data normal distribution and homoscedasticity, respectively. Welch two-sample *t*-tests, and one-way ANOVA and post-hoc Tukey tests were used to compare values among groups of fed and fasted rats. Multiple testing considered calculation of adjusted *p*-values using the Tukey HSD test. Significance was set to *p*-value < 0.05, except for transcriptomics data, where a threshold of 0.001 was considered for adjusted *p*-values. In addition, the false discovery rate according to the Benjamini–Hochberg method was calculated for transcriptomics data.

## 5. Conclusions

The current paper clearly shows a number of regulations at the transcriptional level that are in line with changes in fuel metabolism and the loss of muscle protein/mass during P2 and P3 of prolonged fasting (Figure 7). It should be kept in mind that cell types other than multinucleated fibers reside in skeletal muscles, including satellite cells, immune cells, fibro-adipogenic precursor cells, endothelial cells, and glial cells. These distinct cell type populations express different molecular signatures, as determined recently using a combination of single-cell mass cytometry and single-cell transcriptomics [102]. The changes we report here, which were derived from the analysis of whole muscle isolates, may therefore be influenced by changes in relative proportions of individual cell type populations and their specific response to food deprivation.

Changes at the mRNA level do not always reflect changes in terms of protein abundance or activity. However, the few of them for which we checked matched quite well. Hence, although muscle proteolysis has been shown to be increased essentially during P3 [21], several earlier changes in mRNA levels occurred during P2, which could support the low mobilization of proteins at this stage. Then, an overall induction was observed during P3, including for TGFβ/MSTN, TNFα, and corticosterone signaling pathways; the Smad, NFκB, and EiF2α-ATF4 pathways; and the autophagy and ubiquitin–proteasome systems. The FOXO and AMPK pathways also appeared to be involved, as well as oxidative stress likely induced by fasting. The progressive decline in muscle synthesis rates, which has been reported during prolonged fasting [13], appears supported by less numerous and less pronounced changes during P2 compared to P3. Alteration of mRNA levels notably indicated downregulation of IGF-1/insulin and testosterone signaling pathways; the phosphoinositide 3-kinase (PI3K)/AKT and mTOR pathways; and the processes for transcription, translation, and myogenesis.

It is of note that a number of regulations supposedly in favor of protein synthesis at the level of PI3K/AKT/mTOR pathways, transcription and translations processes, and the response to oxidative stress were observed in both P2 and P3 of fasting. Whether they are actually involved in sparing of muscle proteins during P2 and/or triggered by a higher level of physical activity during P3 due to increased drive for refeeding remains to be determined. They could constitute or reflect important adaptations to limit muscle protein degradation and thus improve nitrogen balance or prepare the body for upcoming refeeding. Future studies should examine whether and to what extent such targets could help develop treatments against muscle wasting and associated pathologies.

## Figures and Tables

**Table 1:** Characteristics of fed and fasted rats.

State	Fed	P2	P3
Main Fuels	Mix *	Lipids	Proteins
**Rat profiles**			
Duration of fasting (days)	-	5.6 ± 0.4	7.8 ± 0.5 ◊
Initial body mass (g)	344.5 ± 1.6	347.7 ± 2.2	346.4 ± 2.4
Final body mass (g)	344.5 ± 1.6	268.5 ± 1.7 #	225.0 ± 2.0 # ◊
Body mass loss (%)	-	22.7 ± 0.4	35.0 ± 0.3 ◊
dm/m.dt (g.100^−1^.d^−1^)	-	3.7 ± 0.3	7.6 ± 0.3 ◊
VO_2_ (mL O_2_.min^−1^.(kg^0.67^)^−1^)	18.95 ± 0.02	14.82 ± 0.7 #	14.44 ± 0.8 #
Respiratory quotient (RQ)	0.92 ± 0.01	0.69 ± 0.01	0.75 ± 0.01
**Plasma**			
NEFA (mM)	0.57 ± 0.04	0.78 ± 0.03 #	0.19 ± 0.02 # ◊
Urea (mg.dL^−1^)	21.4 ± 1.1	16.0 ± 1.1	44.4 ± 3.1 # ◊
Glucose (mM)	8.0 ± 0.3	6.2 ± 0.1 #	5.5 ± 0.3 #
**Muscles**			
TIB mass (mg)	1220.0 ± 47.3	1100.0 ± 20.7	962.0 ± 35.4 # ◊
SOL mass (mg)	330.0 ± 24.5	282.0 ± 13.6	280.0 ± 12.2
Heart mass (mg)	1479.3 ± 70.7	1405.7 ± 41.6	1025.1 ± 47.5 # ◊

Values are the means ± standard error of the mean (SEM) (*N* = 5/group). VO_2_ and the respiratory quotient (RQ) values are those obtained the day of the sacrifice. P2 and P3: phase 2 and phase 3 of fasting; TIB: tibialis anterior muscle; SOL: soleus muscle; NEFA: non-esterified fatty acids. * Main fuels include carbohydrates, lipids and proteins; # *p*-value < 0.05 vs. fed, ◊ *p*-value < 0.05 vs. P2 (one-way ANOVA, Tukey tests, and Welch two sample *t*-tests).

## Supplementary Materials

Supplementary Materials can be found at https://www.mdpi.com/1422-0067/21/17/5984/s1. Table S1: List of the 26535 probes used to compare transcripts expression levels in tibialis anterior muscle of fed and fasted rats. Figure S1: Overview of tibialis anterior transcriptomic response to prolonged fasting in rats.

## Abbreviations

ER	Endoplasmic reticulum
GO	Gene Ontology
miRNA	microRNA
NEFA	Non-esterified fatty acids
OXPHOS	Mitochondrial oxidative phosphorylation
P2	Phase 2 of prolonged fasting
P3	Phase 3 of prolonged fasting
ROS	Reactive oxygen species
RQ	Respiratory quotient
SOL	Soleus muscle
TCA	Tricarboxylic acid cycle
TIB	Tibialis anterior muscle
