# Supplementary material for: Transcriptional Changes Involved in Atrophying Muscles during Prolonged Fasting in Rats
Source: Int J Mol Sci. 2020 Aug 20;21(17):5984. doi: 10.3390/ijms21175984 (PMC7503389; doi:10.3390/ijms21175984)
Supplement: Supplementary file 1 [file ijms-21-05984-s001.zip › Figure S1.pdf]

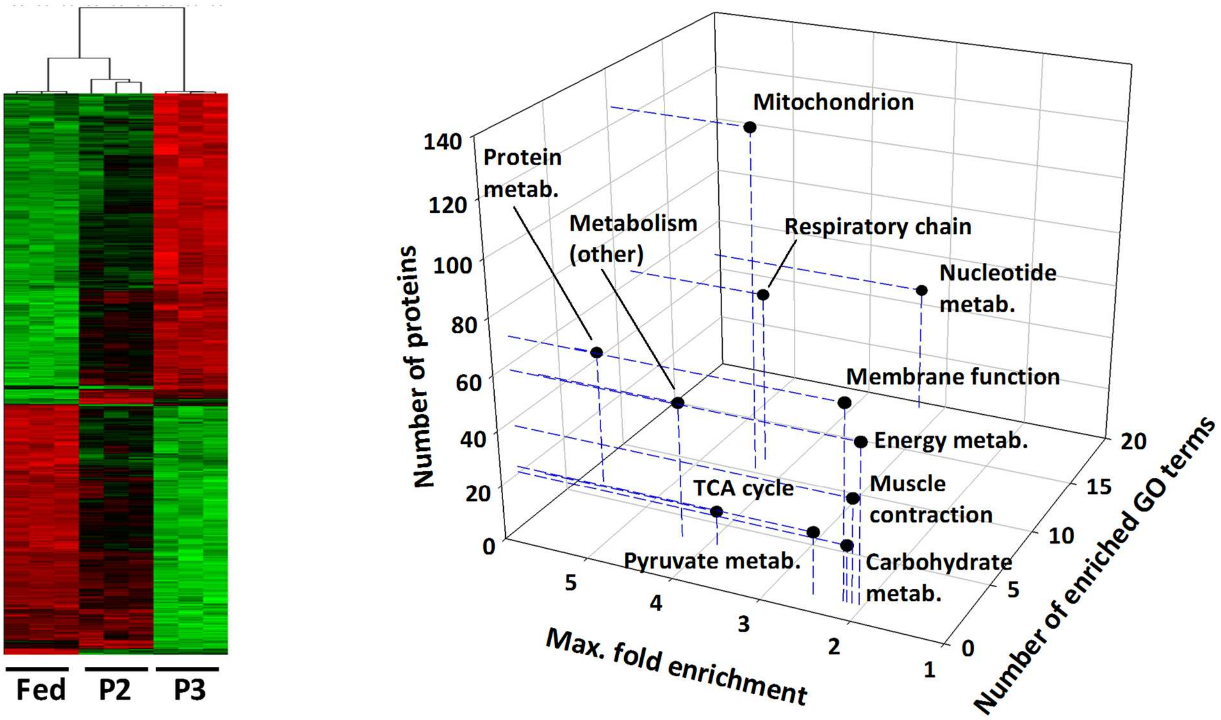

Figure S1. Overview of TIB transcriptomic response to prolonged fasting in rats. The heatmap (left panel) was produced by hierarchical clustering using data from the set of differentially abundant transcripts (ANOVA and Tukey p-value < 0.001, see details in Table S1) in the tibialis anterior (TIB) muscle of rats in the fed state compared to rats in phase 2 (P2) and phase 3 (P3) of fasting (N=3/group). Red, black and green boxes indicate upregulated, intermediate and downregulated transcripts, respectively. Functional annotation analysis from differential transcripts revealed enriched Gene Ontology (GO) terms, which allowed determination of broad functions significantly affected by prolonged fasting (right panel). TCA: tricarboxylic acid.
